# First Detection of *Mycobacterium ulcerans* DNA in Environmental Samples from South America

**DOI:** 10.1371/journal.pntd.0002660

**Published:** 2014-01-30

**Authors:** Aaron Morris, Rodolphe Gozlan, Estelle Marion, Laurent Marsollier, Demetra Andreou, Daniel Sanhueza, Rolland Ruffine, Pierre Couppié, Jean-François Guégan

**Affiliations:** 1 University of Bournemouth, School of Applied Sciences, Dorset, United Kingdom; 2 UMR MIVEGEC IRD-CNRS-Universités de Montpellier 1 et 2, Centre IRD de Montpellier, Montpellier, France; 3 UMR BOREA IRD-MNHN-Université Pierre et Marie Curie, Muséum National d'Histoire Naturelle, Paris, France; 4 Equipe Inserm Avenir ATOMycA, CRCNA INSERM U892 and CNRS U6299, Université et CHU d'Angers, Angers, France; 5 Institut Guyanais de Dermatologie Tropicale, EA 2188, Centre Hospitalier André Rosemon, Cayenne, French Guiana; Universidad Peruana Cayetano Heredia, Peru

## Abstract

The occurrences of many environmentally-persistent and zoonotic infections are driven by ecosystem changes, which in turn are underpinned by land-use modifications that alter the governance of pathogen, biodiversity and human interactions. Our current understanding of these ecological changes on disease emergence however remains limited. Buruli ulcer is an emerging human skin disease caused by the mycobacterium, *Mycobacterium ulcerans*, for which the exact route of infection remains unclear. It can have a devastating impact on its human host, causing extensive necrosis of the skin and underlying tissue, often leading to permanent disability. The mycobacterium is associated with tropical aquatic environments and incidences of the disease are significantly higher on floodplains and where there is an increase of human aquatic activities. Although the disease has been previously diagnosed in South America, until now the presence of *M. ulcerans* DNA in the wild has only been identified in Australia where there have been significant outbreaks and in western and central regions of Africa where the disease is persistent. Here for the first time, we have identified the presence of the aetiological agent's DNA in environmental samples from South America. The DNA was positively identified using Real-time Polymerase Chain Reaction (PCR) on 163 environmental samples, taken from 23 freshwater bodies in French Guiana (Southern America), using primers for both IS2404 and for the ketoreductase-B domain of the *M. ulcerans* mycolactone polyketide synthase genes (KR). Five samples out of 163 were positive for both primers from three different water bodies. A further nine sites had low levels of IS2404 close to a standard CT of 35 and could potentially harbour *M. ulcerans*. The majority of our positive samples (8/14) came from filtered water. These results also reveal the Sinnamary River as a potential source of infection to humans.

## Author Summary

This study provides the first ever recorded extraction of *Mycobacterium ulcerans* DNA from the environment in South America, specifically from French Guiana an ultra-peripheral French territory. *M. ulcerans* is the causative agent responsible for the devastating necrotic skin infection Buruli ulcer, which is prevalent in many tropical countries, notably in western and central Africa, and continues to present outbreaks in the developing world. Despite this, our understanding of the disease remains limited, routes of infection, environmental sources and ubiquity within the environment are still uncertain and only within the past decade have we begun to understand more about this emerging disease.

## Introduction

Buruli ulcer is an emerging human skin disease caused by the mycobacterium, *Mycobacterium ulcerans*. In the same genus as other high profile infectious agents which cause tuberculosis and leprosy, the prevalence of Buruli ulcer has been rapidly increasing in developing parts of the world [1]. Typically found in moist, tropical areas, the disease can have a devastating impact on a human host, causing extensive necrosis of the skin and underlying tissue, often leading to permanent disability. Whilst the exact route of infection remains unclear, the causative mycobacterium is strongly associated with aquatic environments and incidences of the disease are significantly higher on floodplains, or where people come into continual contact with rivers, ponds, swamps and lakes [2], [3]. Since the discovery of specific PCR primers, which are sensitive enough to detect *M. ulcerans* from the environment, it has been found on, or within numerous aquatic species and in environmental samples from aquatic systems [1], [4]–[7]. As the mechanism of infection remains inconclusive, it is not possible to definitely advocate the optimum aquatic conditions necessary for the disease to flourish. We can however speculate on preferred habitats, based on the current evidence and physiological traits. Sequencing of the genome reveals a lack of *crt*I which is responsible for the production of phytoene dehydrogenase, an enzyme that within *M. marinum* is necessary for the synthesis of light-inducible carotenoid pigments [8]. The loss of need for these pigments, which give protection against UV-induced damage, suggests that *M. ulcerans* lives in conditions where it does not require this ability. Culturing *in vitro* has also shown the mycobacterium to have a preference for a low oxygen environment [9]. These traits therefore indicate that the mycobacteria have a preference for environments with low light and oxygen levels.

Identification of the bacteria in the environment has been generally isolated to parts of Australia where there have been significant outbreaks [10]–[12] or tropical regions of Africa where the disease is persistent [4], [5], [13]–[17]. Whilst in South America human cases of the disease have been definitively present since 1969 as for example in French Guiana [1], [18], [19] and previous data suggests the presence of *M. ulcerans* DNA in environmental water sources [18], the DNA has never before been definitively identified in the environment. To ascertain whether environmental conditions where the pathogen is present in French Guiana are comparable to other continents and laboratory analysis extractions of DNA were taken from numerous freshwater bodies in addition to abiotic readings of the water. This has led to the first ever detection of *M. ulcerans* from environmental samples in South America. Further to this we undertook analysis of whether the construction of the Petit-Saut Dam, an hydro-electric installation upstream from an endemic area of French Guiana is correlated with a decline in the number of cases downstream near the city of Sinnamary subsequent to its construction.

## Materials and Methods

**Figure 1:**
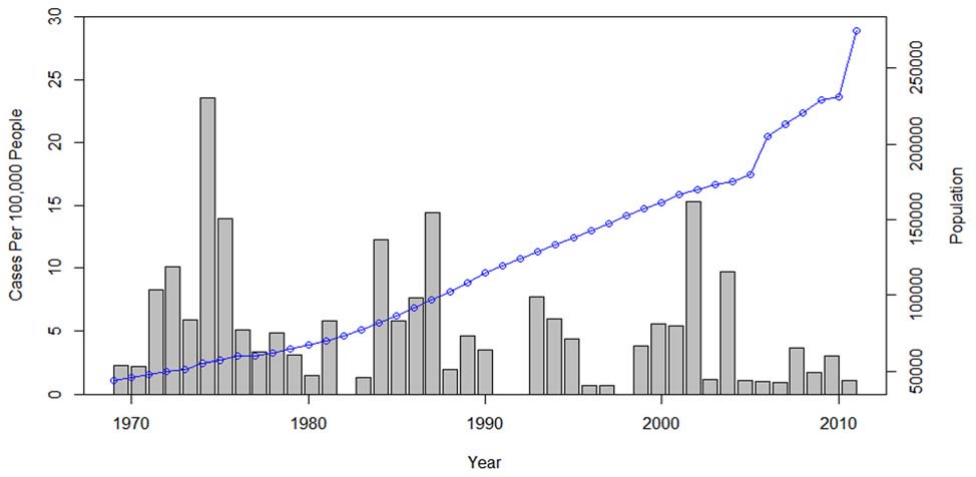
Cases of Buruli ulcer from 1969 to 2012 per 100,000 people, the dotted line represents the increasing human population living in French Guiana.

French Guiana is a French ultra-peripheral territory within South America, bordering the countries of Brazil to the east and south and Suriname to the west. It is 83,534 km^2^ and has a low population density, with most inhabitants residing along a 50 km wide coastal strip dominated by swampy areas. The altitude in the coastal strip is *c.a.* 10–30 m. The rest of the country is almost purely pristine primary tropical rain forest. During the period of 1969 to 2011, 242 cases of Buruli ulcer were reported (Couppie, personal communication) the low population (274,652) makes this number of infections relatively high, with an average number of new notified cases of 2.09/100,000 persons per year (Figure 1).

**Figure 2:**
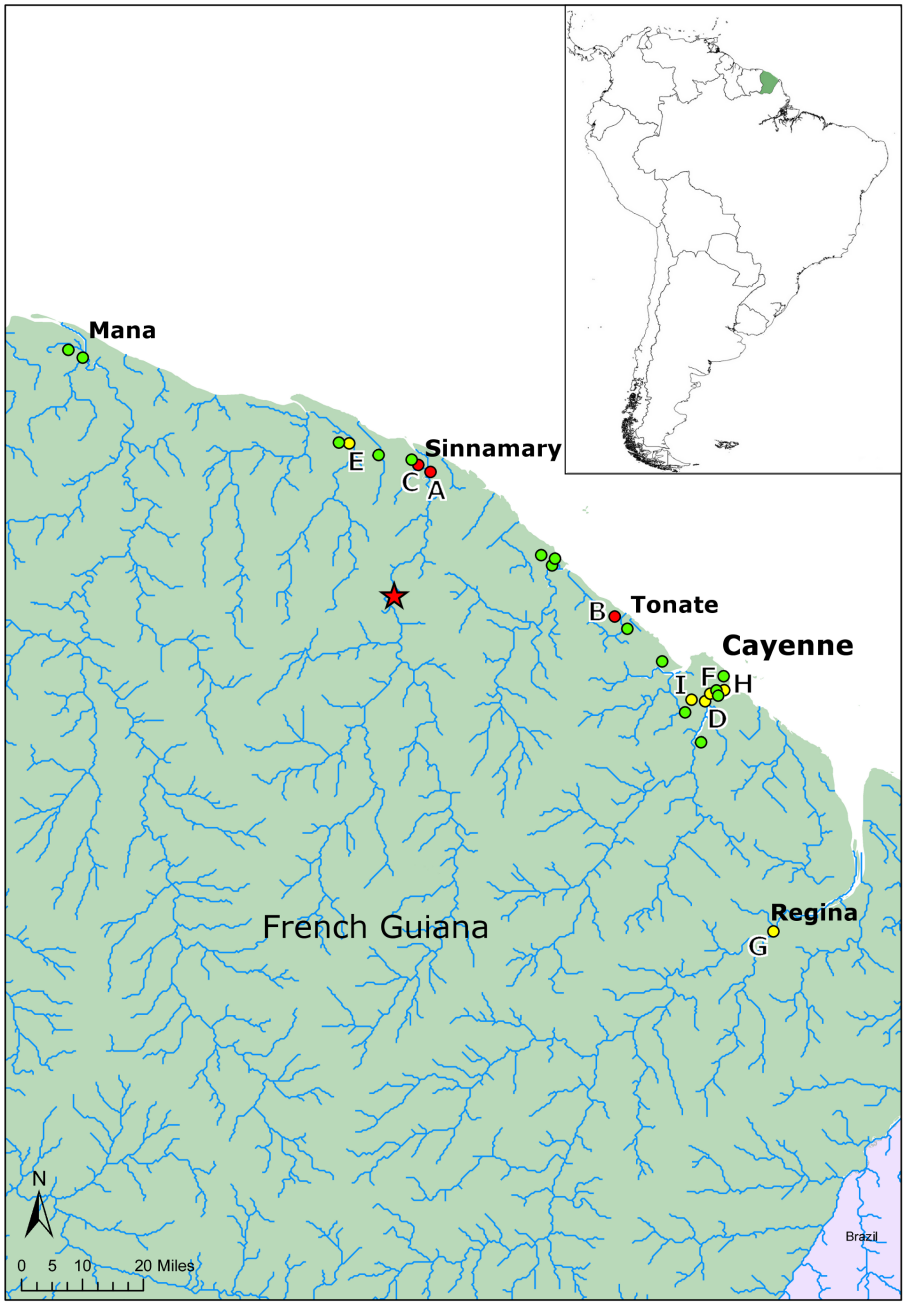
Map showing the approximate location of the twenty-three sampling sites (Text S1). In green are the negative sites to both *Mycobacterium ulcerans* during this survey, in yellow are the site positive to IS2404 alone and in red are the sites positive to both IS2404 and KR. The dam of Petit-Saut built in 1994 is indicated by a star [21].

To identify the presence of *M. ulcerans*, 163 environmental samples were taken from 23 water bodies in French Guiana (Figure 2). Sites where determined by looking for water-bodies in areas where the disease was prevalent and had a high chance of human contact. Indications of this included: trails near or through the site, presence of fishing or boating activities or other recreational uses and close proximity to human settlement. In addition, we looked for similar sites in areas where there have been no cases of disease and sites which had little human contact. Preference was given to sites which remained present during the dry season, as this was indicative of permanence.

Samples taken included: water, soil/sediment and detritus and when present dominant aquatic plant species, algae, biofilms and samples of the semi-aquatic plant *Montrichardia arborescens* (Araceae), a plant species which is characteristic of Amazonian swamps (Table S1 in Text S1). These represent a range of previously described habitats for *M. ulcerans* where positive samples have been identified in other continents [6], [10], [13]. In certain cases when water samples were taken the location contained dense aquatic vegetation, biofilms were unavoidably disturbed from the leaves and stems and it was not possible to take a sample without also capturing biofilms, it is clearly stated in cases where these samples are positive that they contain both water and biofilms.

Water was collected (50 ml) from various sampling points at each site with an attempt to cover all meso-habitats, this generally included: bank-side, the centre of the water body, water from within aquatic vegetation, shaded areas and exposed areas. Water was taken to the laboratory and samples were filtered through 1.6 µmglass microfiber filters before being filtered through 0.4 µm cellulose nitrate membrane and the residue collected and frozen at −20°C for future analysis.

Soil was similarly taken from aquatic areas around the site representing various habitats. Plants, algae and biofilms were collected at various locations when present at the site.

Dissolved oxygen, *pH*, conductivity and water temperature where measured at approximately the locations where each sample was taken.

DNA extraction was carried out using PowerSoil DNA extraction kits (Mo Bio Lab., Carlsbad, USA). Using Real-Time Polymerase Chain Reactions, two primer pairs were used to positively identify the bacillus following standardised methods [10], [20] Table 1.

**Table 1:** Primers and probes for real-time PCR detection.

Primer or probe	Sequence (5′-3′)	Nucleotide positions	Amplicon size (bp)
**IS** ***2404*** ** TF**	AAAGCACCACGCAGCATCT	27746–27762	59
**IS** ***2404*** ** TR**	AGCGACCCCAGTGGATTG	27787–27804	
**IS** ***2404*** ** TP**	6 FAM CGTCCAACGCGATC MGBNFQ	27768–27781	
**KRTF**	TCACGGCCTGCGATATCA	3178–3195	65
**KRTR**	TTGTGTGGGCACTGAATTGAC	3222–3242	
**KRTP**	6 FAM-ACCCCGAAGCACTG-MGBNFQ	3199–3212	

TF, forward primer; TR, reverse primer; TP, probe. Nucleotide position based on the first copy of the amplicon in pMUM001.

### Accession numbers

IS2404: AF003002

KR: BX649209

### Number of cases since the building of Petit-Saut Dam

To help identify the biological niche of *M.ulcerans* in French Guiana we performed a series of statistical tests to identify whether the DNA was predominantly present within certain abiotic conditions. To account of non-normal distributions of data a Wilcoxon-rank sum test was performed on the number of cases in Sinnamary district per month, per 100,000 people for the 18 years since the Dam was constructed and impoundment of water started in January 1994 against the 18 years prior to this period. This was repeated for comparison for cases across the whole of French Guiana.

### Differences between abiotic parameters at sites

To account for non-normal distribution, Wilcoxon tests were used to determine statistical differences between abiotic measurements (*pH*, conductivity, dissolved oxygen and water temperature) at *M. ulcerans* positive sites against the same measurements at negative sites. These tests were also repeated to identify any statistical differences between abiotic factors between sites which were positive for *M. ulcerans*.

## Results

Five samples out of 163 were identified as positive for both IS2404 and KR (CT<35) from three different water bodies in French Guiana; Site A (lat 5.3772, long −52.953883) in Sinnamary (1/19 samples positive), site B (lat 5.3941, long −52.992017) near Tonate (3/5 samples positive) and site C (lat 5.03535, long −52.516483) on Route JoJo a road just outside Sinnamary (1/6 samples positive) (see Table 2 and Figure 2). A further nine sites had low levels of IS2404 close to a standard CT of 35 (Table 3) and could potentially harbour *M. ulcerans*, however it is not possible to conclude this definitively as they were negative for KR.

**Table 2:** Details of positive samples for both IS2404 and KR (CT<35).

Site	Sample Type	Locality	CT Value (IS204)	CT Value (KR)	Bact/ml	Dissolved Oxygen (mg/l)	*pH*	Conductivity (µS/m)	Water Temp (°C)	Season
**A**	Water & Biofilms	Sinnamary	34.70	33.69	18.33	0.74	5.55	197	24.9	Dry
**B**	Water filtrand	Nr Tonate	31.93	33.48	138.9	0.93	6.237	150.8	28.9	Dry
**B**	Water filtrand	Nr Tonate	29.77	30.92	674.6	0.93	6.237	150.8	28.9	Dry
**B**	Water filtrand	Nr Tonate	32.11	34.5	121.8	0.03	5.896	92.4	26.4	Dry
**C**	Water filtrand	Route JoJo	34.14	34.47	27.49	1.93	5.342	32	26.8	Dry

Abiotic parameters taken from where the sample was collected within the water body is also included. Mycobacteria per ml of 50 ml water sample, or 0.25grams of solid sample.

**Table 3:** Details of samples with sites positive for IS2404 (CT>35).

Site	Sample Type	Locality	CT Value	Bact/ml	Dissolved Oxygen (mg/l)	*pH*	Conductivity (µS/m)	Water Temp (°C)	Season
**A**	Water filtrand	Sinnamary	36.2	10.53	0.46	5.3	201	24.7	Dry
**E**	Soil/sediment	Nr Iracoubu	35.41	17.9	NA	NA	NA	NA	Dry
**F**	Soil/sediment	Matoury	37.2	4.877	4.94	6.764	99.50	28.8	Dry
**D**	Water filtrand	Matoury	37.43	2.48	0.05	6.442	14.32	26.6	Dry
**G**	Water filtrand	Regina	36.33	5.553	3.23	5.50	15.6	27.3	Dry
**G**	Water filtrand	Regina	37.39	2.56	0.95	5.35	13.5	26.0	Dry
**G**	Soil/sediment	Regina	35.96	7.297	1.26	5.27	13.9	26.2	Dry
**H**	*M. arborescens* skin	Montjolly	35.53	15.39	0.57	5.62	280	30	Dry
**I**	Filamentous algae	Matoury	36.61	7.33	4.3	4.87	34.8	25.6	Dry

Abiotic parameters taken from where the sample was collected within the water body is also included. Bacteria per ml of 50 ml water sample, or 0.25grams of solid sample.

All three sites (five samples) positive for both IS2404 and KR were typologically similar, with areas that were highly stagnant forming shallow water bodies and with high levels of *M. arborescens* plant growth (Table 2). Dissolved oxygen levels at sites A and B, at the locations where the mycobacterium was found were low (<1 mg/l), at site C dissolved oxygen was higher (1.93 mg/l); however other locations within the site and within a few meters were also similarly low to sites A and C (<1 mg/l). There was no statistical significance for differences in *pH*, conductivity, dissolved oxygen and temperature between positive sites and negative sites.

### Number of cases since the building of Petit-Saut Dam

The number of cases in the 18 years after the construction of the Petit-Saut Dam were significantly lower per 100,000 people per year in Sinnamary at 0.6 than during the 18 years before at 10.1 (Test statistic (W) = 210, p-value = 0.0296), the number of cases in the whole of French Guiana remain statistically similar before and after construction (Test statistic (W) = 132.5, p-value = 0.359).

## Discussion

It is difficult yet to draw definitive conclusions about potential abiotic or biotic factors affecting mycobacterium levels in French Guiana from our current information, whilst our results showed no statistical differences; this may be because of too few positive sites to draw reliable statistical comparisons. Our results extend the range of geographical distribution of environmental *M. ulcerans* to another continent, i.e. south America, where previously the DNA has not been identified in the environment. Similarly we have found that the majority of our positive samples came from filtered water (8/14), which has been the case in other countries [13]. Whilst it could be concluded that the mycobacteria are more prevalent in the water column, this may be an artefact of the sampling methods used. When sampling water we are able to concentrate a large volume onto filters for extraction, whereas with soil, biofilms and plants, the limitations of the extraction kits mean we can only utilise a relatively small fraction of material from a site (0.25 g). In addition the freshwater bodies that we found positive samples in this area were on floodplains, suggesting this category of environment constitutes the source for environmentally-persistent mycobacteria or a “receptacle” concentrating these bacilli from further upstream. From our results we can also recommend the importance of taking multiple abiotic readings from a single site, because we are assessing the ecology of an organism that is living in a microscopic environment, we must consider the large variation in abiotic parameters within a few meters or less in the same water-body (for example Site B, Tables 2 and 3).

The low levels of cases combined with a low population density in a territory such as French Guiana suggest identification from the environment would be difficult; however we were able to find positive sites with less than 200 samples, which in themselves are relatively small components of a system. This would reinforce the possibility that *M. ulcerans* is a fairly common and widely distributed mycobacterium, and it is other factors, e.g. socio-economic (i.e. levels of human contact with water, sanitation etc), transmission related (i.e. presence of potential vectors, hosts or reservoirs), or habitat modifications (deforestation, dam construction, etc), that might be the primary drivers of cases.

The finding of the majority of positive sites from the area around Sinnamary River downstream to the dam is of interest, the first cases of Buruli ulcer in French Guiana were recorded here and approximately 10% of human cases of Buruli ulcer in French Guiana concern the inhabitants of the Sinnamary. In the Sinnamary region the number of cases has been very low since 1994, with significantly less cases in the 18 years after 1994, despite an increasing human population. Whilst changes in the behaviour of people may have an influence, the building of the Petit-Saut Dam (Figure 2) may also be playing a role. The dam has profound effects on the level of water which comes into the area, possibly reducing flooding or regulating water flows, or potentially limiting mycobacteria being brought upstream from the rainforest and riverine swamp areas.

The results presented here suggest there remains a potential for future infection in French Guiana, as our knowledge of transmission improves we hope to be able to identify at which point a water body harbouring the bacteria can become a site of transmission to people. They also provide a basis for future studies of *M. ulcerans* in South America.

## Supporting Information

Text S1 Supporting information includes details of the number and type of samples taken for all sites.
(DOC)
