# Supplementary material for: First Detection of Mycobacterium ulcerans DNA in Environmental Samples from South America
Source: PLoS Negl Trop Dis. 2014 Jan 30;8(1):e2660. doi: 10.1371/journal.pntd.0002660 (PMC3907311; doi:10.1371/journal.pntd.0002660)
Supplement: Text S1 — Supporting information includes details of the number and type of samples taken for all sites. (DOC) [file pntd.0002660.s001.doc]

**Text S-1**

**Table S-1 Site locations and the number and type of samples taken. In bold both IS2404 and KR identified.**

| **Site** | **Latitude** | **Longitude** | **Location** | **IS2404** | **KR** | **Total Samples** | **Soil** | **Water** | **Algae/**  **Biofilms** | **Plant Material** | ***M. arborescens*** | **Detritus** | **Insect** |
| --- | --- | --- | --- | --- | --- | --- | --- | --- | --- | --- | --- | --- | --- |
| **A** | **5.3772** | **-52.953883** | **Sinnamary** | **+** | **+** | **19** | **3** | **13** | **1** | **-** | **-** | **1** | **1** |
| **B** | **5.03535** | **-52.516483** | **Nr Tonate** | **+** | **+** | **5** | **-** | **3** | **2** | **-** | **-** | **-** | **-** |
| **C** | **5.3941** | **-52.992017** | **Route Jojo** | **+** | **+** | **6** | **-** | **6** |  | **-** | **-** | **-** | **-** |
| D | 4.834467 | -52.3021 | Matoury | + | - | 6 | - | 6 | - | - | - | - | - |
| E | 5.44505 | -53.158183 | Nr Iracoubo | + | - | 5 | 2 | 3 | - | - | - | - | - |
| F | 4.83365 | -52.3004 | Matoury | + | - | 18 | 4 | 8 | 3 | - | - | 3 | - |
| G | 4.300417 | -52.13995 | Regina | + | - | 6 | 3 | 2 | 1 | - | - | - | - |
| H | 4.8608 | -52.25675 | Montjoly | + | - | 9 | 1 | 3 | 2 | 1 | 1 | 1 | - |
| I | 4.838083 | -52.35325 | Matoury | + | - | 15 | 4 | 4 | 3 | 3 | 1 | - | - |
| J | 5.6666 | -53.7799 | Mana | - | - | 8 | 2 | 6 | - | - | - | - | - |
| K | 5.652183 | -53.825 | Mana | - | - | 4 | - | 3 | 1 | - | - | - | - |
| L | 5.44505 | -53.165 | Nr Iracoubo | - | - | 5 | 1 | 3 | 1 | - | - | - | - |
| M | 5.428733 | -53.088717 | Nr. Sinnamary | - | - | 3 | - | 3 | - | - | - | - | - |
| N | 4.729714 | -52.318397 | Roura | - | - | 12 | 4 | 7 | 1 | - | - | - | - |
| O | 5.1561 | -52.665233 | Kouru | - | - | 3 | - | 3 | - | - | - | - | - |
| P | 5.172417 | -52.658467 | Kouru | - | - | 5 | - | 3 | 2 | - | - | - | - |
| Q | 5.180617 | -52.66165 | Kouru | - | - | 4 | 1 | 2 | 1 | - | - | - | - |
| R | 5.403217 | -52.99575 | Route Jojo | - | - | 1 | - | 1 | - | - | - | - | - |
| S | 5.00615 | -52.4869 | Tonate | - | - | 5 | 1 | 4 | - | - | - | - | - |
| T | 4.929067 | -52.403817 | Macouria | - | - | 7 | - | 5 | 2 | - | - | - | - |
| U | 4.893783 | -52.257883 | Montjolly | - | - | 3 | - | 3 | - | - | - | - | - |
| V | 4.833183 | -52.2993 | Matoury | - | - | 5 | 1 | 2 | - | - | - | 2 | - |
| W | 4.837767 | -52.349483 | Matoury | - | - | 6 | 2 | 2 | 2 | - | - | - | - |
| X | 4.860267 | -52.2753 | Montjolly | - | - | 3 | - | 3 | - | - | - | - | - |
